# Endoscopic endonasal technique: treatment of paranasal and anterior skull base malignancies

**DOI:** 10.5935/1808-8694.20130138

**Published:** 2015-10-08

**Authors:** Pornthep Kasemsiri, Daniel Monte Serrat Prevedello, Bradley Alan Otto, Matthew Old, Leo Ditzel Filho, Amin Bardai Kassam, Ricardo Luis Carrau

**Affiliations:** a M.D. (Department of Otorhinolaryngology, Srinagarind Hospital, Faculty of Medicine, Khon Kaen University, Khon Kaen, Thailand); b M.D. (Department of Neurological Surgery, Wexner Medical Center, The Ohio State University, Columbus, OH, USA); c M.D. (Department of Otolaryngology-Head and Neck Surgery, Wexner Medical Center, The Ohio State University, Columbus, OH, USA); d M.D. (Department of Neurological Surgery, Ottawa University, Ottawa, Canada); Institute Wexner Medical Center, at The Ohio State University

**Keywords:** endoscopy, neoplasms/surgeries, skull base/injuries

## Abstract

Technical and technological innovations have spearheaded the expansion of the indications for the use of endoscopic endonasal approaches to extirpate malignancies of the sinonasal tract and adjacent skull base.

**Objective:**

Critical review of the available literature regarding the use of endoscopic endonasal approaches including indications, limitations, surgical techniques, oncologic outcome, and quality of life.

**Method:**

Various endoscopic endonasal techniques are reviewed according to the origin and local extension of sinonasal and skull base malignancies including anterior cranial base, nasopharynx, clivus, and infratemporal fossa. In addition, the available literature is reviewed to assess outcomes.

**Conclusion:**

Endoscopic endonasal approaches are an integral part of the armamentarium for the treatment of the sinonasal tract malignancies and skull base. In properly selected cases, it affords similar oncologic outcomes with lower morbidity than traditional open approaches. Nonetheless, these minimal access approaches should be considered a complement to well-established open approaches, which are still necessary in most advanced tumors.

## INTRODUCTION

Sinonasal carcinomas are uncommon malignancies, representing approximately 0.2% of all cancers and 3% to 5% of cancers in upper aerodigestive tract1., 2., 3.. In their early phase, these tumors commonly produce symptoms that are similar to those caused by inflammatory sinonasal disease: nasal airway obstruction, epistaxis, headache, facial pain, and nasal discharge. Lack of any significant symptom is also relatively common; thus, causing a delay in diagnosis and progression of the tumor to advanced stages. Optimal treatment remains undefined, however; surgery followed by postoperative radiotherapy or chemoradiotherapy is currently considered for the vast majority of patients with locally advanced disease.

Oncologic surgery implies an adequate resection (complete) of the tumor sparing normal structures. En bloc resection of sinonasal tumors, considered standard treatment for many years, flourished with the development of transfacial approaches (i.e. lateral rhinotomy, and midfacial degloving) and the combination of transcranial and transfacial approaches for tumors involving the skull base. Open approaches, however, are associated to postoperative morbidity that includes external scars, maxillofacial cosmetic defects due to ostectomies or translocation of the maxillofacial skeleton; and, more important, injury related to retraction of the brain. Alternative minimal invasive techniques have been progressively introduced to reduce or avoid these complications.

Over the past two decades, endoscopic endonasal techniques have been developed mainly based on an expanded fund of knowledge regarding endoscopic anatomy, and the development of novel surgical techniques and technologies that have enabled the complete resection of malignancies (i.e. with negative margins). Endoscopic endonasal approaches to the skull base comprise two critical tenets, bilateral nasal access to allow for a two-surgeon-four-hand technique (i.e. bimanual dissection and dynamic movement of the endoscope) and customized removal of bone to create a wide surgical corridor that allows adequate visualization and instrumentation.

A significant advantage of the endoscopic endonasal approaches is that they provide the most direct access to the ventral skull base while obviating the retraction and manipulation of critical neurovascular structures4., 5., 6., 7.. To facilitate their understanding and communication among surgeons, the endoscopic endonasal approaches can be organized according to their anatomical orientation into sagittal (median) and coronal planes. Approaches in the sagittal plane access the ventral skull base from the frontal sinus to the second cervical vertebra^8^; therefore, providing an endoscopic corridor for an oncologic resection the anterior cranial base, median nasopharynx, and clivus. Endoscopic approaches in the coronal plane expose lesions that extend laterally to the midline of the roof of the orbit (anterior coronal approaches), the floor of the middle cranial fossa (middle coronal approaches), and the jugular foramen (posterior coronal approaches)^9^.

Unfortunately, advances in the reconstruction after endoscopic endonasal approaches lagged significantly behind advances in the approach and resection of tumors of the skull base. Reconstructive outcomes were variable until the adoption of the Hadad-Bassagaisteguy nasoseptal flap, which is a robust flap that can cover an area comprising from the posterior wall of the frontal sinus to the sellae turcica and from orbit to orbit10., 11., 12.. Other alternative flaps were subsequently described, specifically used in patients in whom the nasoseptal flap is unavailable due to invasion of tumor or previous surgery^13^. Alternative pedicled flaps include turbinate flaps14., 15., 16., transpterygoid temporoparietal fascia flap^17^, transfrontal pericranial flap18., 19., Oliver palatal flap20., 21., lateral nasal wall flaps22., 23..

In this manuscript, we will present our view of the current principles and techniques for the endoscopic endonasal resection of malignancies involving specific sites of the skull base. No surgical technique should be discussed in a vacuum, as its outcomes are highly dependent on an adequate selection of patients, thorough planning, precise surgical technique and use of adjunctive measures; therefore, we present a brief discussion of our preoperative and intraoperative preparations and management. In addition, we will present a critical appraisal of the available literature, as well as our experience.

## PREOPERATIVE EVALUATION

Preoperative evaluation for patients undergoing an endoscopic endonasal resection does not differ from that of other approaches. All patients undergo a complete examination of head and neck with emphasis on the sinonasal region, orbit, status of cervical lymph nodes, and basic neurologic function (especially cranial nerve status). In addition, sinonasal endoscopy provides a detailed assessment of the sinonasal tract and tumor characteristics, ascertaining any anatomical variation, absence of active infection, vascularity of the tumor and occasionally its site of origin.

Computed tomographic scan (CT) and magnetic resonance imaging (MRI) are complementary investigations that evaluate the bony and soft tissue extensions of the tumor, including orbital, intracranial, perineural, and vascular extensions, and suggest its degree of vascularity; therefore, serving as preoperative surgical maps. For tumors that involve or are adjacent to critical neurovascular structures, we advocate a CT angiography (CTA) for surgical planning, and for image fusion with MRI during intraoperative navigation.

It is critical to confirm the tumor histology before the definitive surgery. However, there are exceptional circumstances that justify a deviation from this axiom. Pathognomonic characteristics in the imaging of the tumor, patient comorbidities increasing the surgical risk (i.e. a second surgery maybe ill advised), and organizational or institution logistics may warrant special consideration to using intraoperative histological confirmation followed by definitive resection.

Inflammation and bleeding associated with a biopsy may alter the tumor appearance in the MRI; thus, we prefer completing the imaging first, whenever possible. In addition, a contrasted imaging may afford an estimate of the tumor vascularity, and cue the team to prepare for the possibility of significant bleeding. In general, large tumors that are easily visualized in the anterior nasal cavity may be biopsied with minor risk in the office setting. Other tumors are commonly biopsied in the operating room where an adequate volume of tissue can be sampled, and where bleeding and possible communication with cerebrospinal fluid (CSF) can be avoided or managed promptly and effectively.

A fused PET and CT scan is best to identify metastasis in patients presenting with advanced disease, and those who present with tumors that are known to spread hematogenously (e.g. sarcomas, melanoma, and adenoid cystic carcinoma). Similarly, sarcomas and other high-grade malignancies with dural transgression warrant a cytological analysis of the cerebrospinal fluid (i.e., lumbar spinal puncture), and a MRI of the spine to rule out the presence of “drop metastasis”^24^.

## SURGICAL SETUP

After orotracheal intubation, the endotracheal tube is fixed toward the left side and the patient is placed in a 3-pin head holder with the neck tilted slightly to the left and turned slightly to the right. In select cases, a “horseshoe” type head holder can be used; however, we prefer to pin the head in prolonged surgeries, those in which extensive drilling is anticipated and those in which the patient cannot be paralyzed due to the monitoring of cranial nerves (i.e. EMG monitoring).

The nose is decongested with topical 0.05% oxymetazoline or epinephrine 1/10,000–1/20,000. Povidone is applied to the perinasal and periumbilical regions (in the event that an autologous fat free graft is required for reconstruction). If the patient presents a high risk for internal carotid artery (ICA) injury, or if the surgeon anticipates the need for fascia lata free grafting, the thigh is also prepped. Broad-spectrum prophylactic perioperative antibiotics (third generation cephalosporin with cerebrospinal fluid penetration) are administered and continued through the second postoperative day (nasal packing may require an extended course of antibiotics).

Monitoring of the somatosensory evoked potentials (SSEPs) identifies early signs of brain compromise due to ischemia, edema, contusion and hemorrhage; thus, we advocate its use when available. In addition, cranial nerve injuries may occur when resection of tumor extends into the retrobulbar orbit, superior orbital fissure, or cavernous sinus; therefore, monitoring with electromyography of the pertinent cranial nerve and muscle is indicated.

A 0° rod lens endoscope provides undistorted and adequate visualization of the surgical field and enables the use of straight instrumentation (its line of sight matches the geometry of the instruments). Conversely, angled lens endoscopes allow looking around the corner; however, they are more difficult to use due to their distorted view and because they require angled instruments to match their line of sight. Therefore, we prefer to use the 0° rod lens endoscope coupled to a high definition endoscopic camera and monitor for visualization during most of the surgery, maintaining a clean lens by manual irrigation.

## ENDOSCOPIC ENDONASAL RESECTION

### General Principles

As previously mentioned, the sinonasal tract is a somewhat silent region in the sense that symptoms are mild or absent until the tumor attains significant extension; therefore, most patients present with advanced carcinomas that often involves the cranial base or extends laterally to the infratemporal fossa. Endoscopic endonasal approach can address the target lesions via modular approaches anatomically oriented in the sagittal plane or coronal plane. However, pure endoscopic endonasal approaches for malignant tumors are limited by paramedian critical neurovascular structures (i.e. orbit, optic nerve, ICA); thus, extension beyond these lateral boundaries constitute a contraindication for an endoscopic endonasal approach (or indicate the need for a second external approach). Other similar contraindications include tumor extending laterally across the mid-orbital plane, invasion of the orbital soft tissues (i.e. requiring an orbital exenteration), involvement of anterior table or lateral recesses of the frontal sinus, the need to perform a total maxillectomy, or the need to remove skin due to tumor invasion. Furthermore, an active sinonasal bacterial or fungal infection contraindicates an elective endoscopic endonasal transdural approach; thus, it should be treated prior to surgery25., 26..

Surgery aims toward a complete tumor extirpation with negative margins. However, opposed to traditional open approaches, endoscopic endonasal resection of a malignancy is rarely completed en bloc. This salient feature of endoscopic endonasal resection has been criticized by some; however, a similar dilemma has been previously faced with cancers in other regions of the head and neck including pharyngeal and laryngeal squamous cell carcinoma, and skin cancer (Moh's surgery). Select cancers in these areas are currently resected in a piecemeal or layered fashion without jeopardizing the outcomes. Similar findings have been reported for the resection of sinonasal malignancies with endoscopic endonasal surgery, suggesting that a piecemeal or layered resection does not compromise the oncologic resection^27^.

### Endoscopic endonasal anterior cranial base resection

#### Indications and limitations

Sinonasal carcinomas (e.g. squamous cell carcinoma, melanoma, basal cell carcinoma, adenoid cystic carcinoma, and mucoepidermoid carcinoma) are the most common malignancies to involve the anterior cranial base. In addition, several primary malignancies originate from olfactory epithelium and other neural tissue of the anterior cranial base (e.g. esthesioneuroblastomas, neuroendocrine carcinomas, sinonasal undifferentiated carcinomas and primitive neuroectodermal tumors). Lymphoreticular tumors and sarcomas (e.g. lymphomas, plasmacytomas, chondrosarcomas, osteosarcomas, rhabdomyosarcomas, malignant hemangiopericytomas, and malignant giant cell tumors) are also encountered, although less commonly. Many of these tumors are median lesions that can be addressed via an endoscopic endonasal approach. However, as previously mentioned, a pure endoscopic endonasal approach has important limitations. A traditional open or endoscopic-assisted craniofacial resection should be considered when the tumor extends beyond the boundaries that are amenable to an endoscopic resection. However, if the tumor is considered unresectable a pure endoscopic endonasal approach may play a palliative role to open the sinonasal airway, provide drainage to the paranasal sinuses, control hemorrhage or decompress the orbit, or other neural structures^27^.

#### Surgical technique (Figure 1)

Identification of the site of origin is important but may not by reliably established until surgery. This is true for the great majority of patients, who present with advanced, large tumors that occlude the nasal cavity. Therefore, surgery typically starts with a debulking of the tumor to define the site of origin, as well as its relationship with the skull base and other critical anatomy. The initial approach is usually performed through the nostril with predominant involvement of tumor; thus, facilitating its debulking. An ipsilateral middle turbinectomy gains further space for instrumentation and allow a better assessment of the tumor. If the tumor involves the nasal cavity bilaterally, both middle turbinates are resected. A wide sphenoidotomy and nasoantral window allow the identification of the skull base (i.e. roof of the sphenoid sinus) and lamina papyracea.

**Figure 1:**
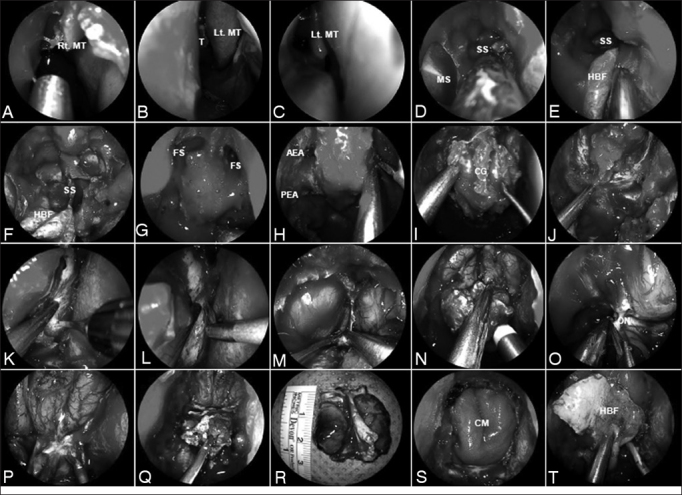
A-C: Anterior skull base tumor involving the nasal cavity bilaterally; therefore, requiring a bilateral resection; D: Sequentially, a wide sphenoidotomy and nasoantral window are completed; E-F: A HBF is raised on the healthy side of the nasal septum and is stored in the nasopharynx; a bony posterior septectomy and Caicedo's reverse flap follows. This allows communicating the posterior nasal cavities into a single chamber; G: Bilateral frontal sinusotomy (Draf Type III), followed by the drilling of the crista galli, help to establish the anterior resection margin; H: The anterior and posterior ethmoid arteries are coagulated and divided; I: Anterior cranial base bone is dissected off the dura including fovea ethmoidalis, cribriform plate, and crista galli; J-L: After complete bony removal, the dura is exposed and opened in the ‘inverted U′, starting the incisions at the lateral margins; M-N: The cerebral falx is incised in a ventro-caudal direction; O: The olfactory nerves are resected for margins and their stumps are cauterized; P-R: This allows the infero-posterior displacement and resection of any residual tumor and cribiform plate en bloc; S: Once margins are deemed free from tumor by histological analysis, the reconstruction is performed; T: A collagen matrix graft is inlaid and the HBF is onlaid. Rt. MT: Right middle turbinate; Lt. MT: Left middle turbinate; MS: Maxillary sinus; SS: Sphenoid sinus; HBF: Hadad-Bassagaisteguy nasoseptal flap; FS: Frontal sinus; AEA: Anterior ethmoidal artery; PEA: Posterior ethmoidal artery; CG: Crista galli; ON: Olfactory nerve; CM: Collagen matrix.

A Hadad-Bassagaisteguy flap may be raised from the contralateral side and stored in the sphenoid, ipsilateral antrum, or left flat against its ipsilateral lateral nasal wall according to the need for exposure of each of sites. A subsequent posterior bony septectomy and Caicedo's reverse flap (CRF)28., 29. follows. We recommend histological analysis (negative margins) of the superior and posterior incisions (or any other pertinent area) of both flaps to avoid leaving persistent tumor, or seeding the tumor into new areas such as the columella. Resection of the nasal septum is tailored to the extension of the disease. Therefore, bilateral extension often mandates a large septectomy that precludes the harvest of the pedicle nasoseptal flap. Similarly, if the tumor has contralateral involvement of rostrum of the sphenoid or pterygopalatine fossa, these flaps will not be available and an alternative reconstruction should be planned.

Bilateral ethmoidectomies further expose the superior aspect of the nasal cavity and the attachments of the turbinates and septum to the anterior cranial base. Resection of the superior turbinate while completing the posterior ethmoidectomy, exposes the spheno-ethmoid recess and natural ostium of the sphenoid sinus that may be enlarged to allow inspection of the sinus, enhance drainage, or remove tumor. The nasofrontal recess is identified and a Draf 2 (for a unilateral resection) or Draf 3 (for a bilateral resection) is opened. These steps are repeated bilaterally according to the extension of the tumor.

Removal of the lamina papyracea offers an additional margin and aids in the identification of the anterior and posterior (and in some patients a middle) ethmoidal neurovascular bundles. The remaining tissue includes the tumor base, which is removed before drilling the bone of the anterior fossa floor. A frontal sinusotomy (unilateral Draf 2 or a Draf 3), followed by the drilling of the crista galli, help to establish the anterior resection margin.

The fovea ethmoidalis and cribriform plate are drilled thin and dissected off the dura bilaterally. If necessary due to tumor extension, the bone of the planum sphenoidale may be removed back to the level of the optic nerve foramina. The anterior and posterior ethmoid arteries are identified, coagulated with a bipolar electrocautery, and divided. After completing the bony removal, the dura is exposed and opened in the ‘inverted U’ fashion, starting at the lateral margins to avoid injury of the median fronto-polar, fronto-orbital and parafalcine arteries. The cerebral falx is incised in a ventro-caudal direction under direct visualization. This allows infero-posterior displacement of the residual tumor and the dura en bloc. Using gentle suction as traction, and sharp dissection, all arachnoid adherences are freed and both olfactory bulbs are resected for margins. Great care is taken to preserve the fronto-polar arteries and the integrity of the frontal lobes.

The final defect frequently extends from orbit to orbit and from crista galli to planum sphenoidale. An important olfaction-sparing variation may be possible in patients with select unilateral tumors. In this technique the olfactory cleft as well as the middle and superior turbinates of the uninvolved side are preserved.

#### Reconstructive technique

Once adequate resection margins are confirmed by intraoperative histological analysis, and hemostasis is corroborated, we proceed with the reconstruction of the defect using an inlay sheet of collagen matrix or fascia lata. Then, the Hadad-Bassagaisteguy flap is retrieved and positioned over the defect. Occasionally, a fat graft is inserted prior to positioning the flap; this measure obliterates dead space (i.e. sphenoid sinus); therefore, optimizing the flap position and increasing its anterior reach. Finally, the reconstruction is bolstered with nasal packing.

In patients where the Hadad-Bassagaisteguy flap is not available (due to tumor invading the nasal septum, sphenoid rostrum or pterygopalatine fossa, or previous surgical resection of the septum), a pericranial flap should be considered18., 19.. The scalp hair is parted following the planned coronal incision but no hair is shaved. After sterile preparation and injection with lidocaine 1% with epinephrine 1/100,000 a coronal incision is carried down to the calvarium (between the temporalis muscles) or down to the superficial layer of the deep temporal fascia (over the temporalis muscles). Alternatively, a subgaleal dissection, posterior to the coronal incision may be used to increase the length of the pericranial flap. The scalp is elevated from the skull in a caudal direction to reach the orbital rims, and to identify the supraorbital and supratrochlear neurovascular bundles. Once the neurovascular bundles have been dissected from their corresponding notches or foramina, the scalp dissection continues caudally to expose the nasion and nasal bones. The loose areolar tissue and periosteum (i.e. pericranium) are subsequently elevated from the galea (unilateral) following a cephalo-caudal direction and narrowing down to about 3 cm at the level of the pedicle (unilateral).

A high-speed drill with a 4 mm cutting or coarse diamond burr is used to open a window at the level of the nasion into the nasofrontal recesses. The pericranial flap is then transposed through the window under endoscopic visualization, preventing torsion of the vascular pedicle. The pericranial flap is gently pressed down and flattened to cover the inlay fascial graft, exposed dural edges, and the surrounding bony framework of the defect with an extradural extracranial onlay technique.

#### Endoscopic endonasal transpterygoid extension

Tumors involving the nasopharynx, pterygopalatine (PPF) or infratemporal fossa (ITF), require resection via a transpterygoid corridor. The initial steps of the approach are usually performed through the predominantly involved nostril. An ipsilateral ethmoidectomy, wide sphenoidotomy and medial maxillectomy (with or without an anterior extension or an endoscopic Denker's approach) create a large “single chamber” corridor that allows unimpeded lateral visualization and instrumentation.

**Figure 2:**
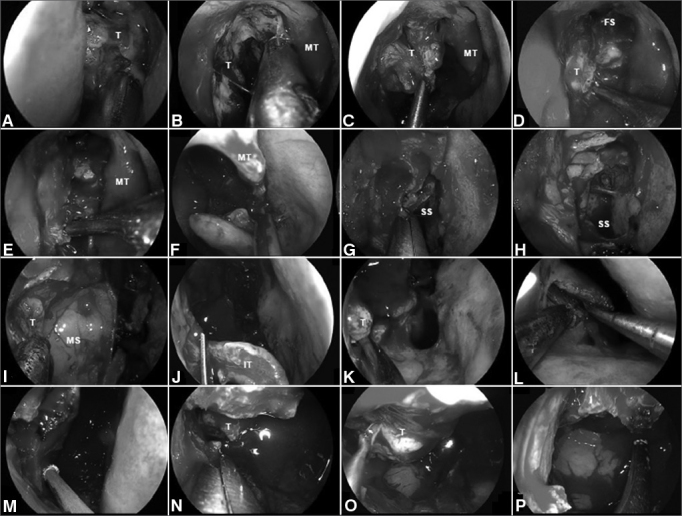
A-B: Intraoperative photographs demonstrating the resection of a right ethmoidal adenocarcinoma. Firstly, the tumor is debulked to identify it origin; C-D: Subperiosteal dissection of the tumor separates it from the medial orbital wall, nasoethmoidal complex and nasofrontal recess; E: The dissection continues along the maxillary line and posteriorly, along the lamina papyracea, to reach sphenoid sinus; F: The left middle turbinate is removed to establish an adequate margin and to expand the space for instrumentation; G-H: Wide sphenoidotomies establish the posterior margin; I: Residual tumor at the anterior aspect of the medial wall of the maxillary sinus cannot be adequately removed via a midmeatal antrostomy; J: An endoscopic medial maxillectomy is performed with the resection of the inferior turbinate; K: The resection extends from the orbit down to the floor of nose; however, its' exposure is insufficient. Therefore, an endoscopic Denker's approach is deemed necessary for a full exposure; L: The piriform aperture and ascending process of the maxilla are removed, dissecting the nasolacrimal duct and transecting it sharply. Exposure of the piriform aperture requires a vertical incision on the edge of the aperture; M-N: This edge can be palpated with a blunt dissector to optimize the placement of the incision, which is then carried through the periosteum down to bone. A subperiosteal lateral dissection exposes the anterior maxilla. The medial maxillectomy is then extended to remove the piriform aperture and sufficient anterior maxillary wall to expose the entire confines of the antrum; O-P: This corridor facilitates the adequate resection of tumor with negative margins; T: Tumor; MT: Middle turbinate; FS: Frontal sinus; SS: Sphenoid sinus; MS: Maxillary sinus; IT: Inferior turbinate.

Specifically, a transpterygoid approach requires a minimum of a wide midmeatal naso-antral window (NAW) that should be maximized extending the opening antero-posteriorly, from the nasolacrimal duct to the posterior antral wall and cephalo-caudally from the floor of the orbit to the superior aspect of the inferior turbinate. This midmeatal naso-antral window adequately addresses lesions above the vidian canal. Malignancies, however, often extends below the vidian canal; thus, an appropriate transpterygoid approach will demand an endoscopic medial maxillectomy extending from the inferior orbital wall to the floor of the nasal cavity and from the nasolacrimal duct to the posterior wall of the antrum. This exposes the entire height of the posterior wall even using a 0° rod lens endoscope. Occasionally, the medial maxillectomy needs to be extended anteriorly to provide enhanced lateral exposure for extensive tumors in the infratemporal fossa. This may even require an endoscopic Denker's approach, which includes the removal of the remaining inferior turbinate, anterior aspect of the inferior meatus and anterior wall of the maxilla (Figure 2). Exposure of the piriform aperture requires a vertical incision just anterior to the head of the inferior turbinate, over the edge of the aperture. This edge can be palpated with a blunt dissector to optimize the placement of the incision, which is then carried through the periosteum to facilitate a subperiosteal exposure of the anterior maxilla. The medial maxillectomy is then extended anteriorly to remove the piriform aperture and sufficient anterior maxillary wall to expose the entire lateral wall of the antrum.

An anterior extension of the medial maxillectomy may be tailored to the specific visualization need, e.g. confining it to removal of the lateral wall inferior to the nasolacrimal opening. However, a full Denker's approach includes removing the anteromedial aspect of the ascending process of the maxilla (piriform aperture), dissecting and transecting the lacrimal duct sharply. This allows a line of sight that extends to the most lateral aspect of the infratemporal fossa.

Following a wide midmeatal naso-antral window or a medial maxillectomy, the mucoperiosteum covering the superior third of the posterior wall of the antrum and the corresponding level over the perpendicular plate of the palatine bone is elevated to identify the sphenopalatine foramen. Using 1–2 mm Kerrison's rongeurs, the anterior aspect of the sphenopalatine foramen can be removed to expose the pterygopalatine fossa. Bone removal is extended inferiorly to reach the level of the antral floor, and laterally to reach the inferior orbital fissure. Cautious dissection of the medial aspect of the posterior wall of the maxillary sinus is necessary, as it forms part of the descending palatine canal containing the greater palatine nerve and descending palatine artery. After the exposure is attained, the tumor can be removed from the pterygopalatine fossa, identifying and controlling the internal maxillary artery.

Extension of the tumor into the middle cranial base or infratemporal fossa mandates the resection of pterygoid process to provide adequate access to the tumor. Initially, the ipsilateral sphenoidotomy is extended laterally, opening the lateral recess of the sphenoid sinus. When tumor extends to the middle cranial base (i.e. petrous apex or Meckel's cave), it is essential to drill the sphenoid sinus floor until it becomes flush with the level of the clival recess. Subsequently, the vidian canal is dissected until the transition from paraclival (vertical) to petrous ICA (horizontal) is identified visually and confirmed with an acoustic Doppler sonography. One should proceed with removing the tumor from petrous apex and Meckel's cave only after the exposure (i.e. removal of the pterygoid base) is deemed adequate.

To enter the infratemporal fossa, the trunk of the internal maxillary artery is divided at the pterygomaxillary fissure, whereas the vidian neurovascular bundle, and the branch of the sphenopalatine ganglion to CN V_2_ are divided just distal to their respective foramina. This exposes the entire height of the anterior aspect of the pterygoid process. A subperiosteal elevation avoids bleeding from the pterygoid plexus while dissecting the lateral and medial pterygoid muscles; which are detached before drilling out the lateral pterygoid plate. After complete removal of the pterygoid process, the infratemporal fossa can be accessed to extirpate the tumor. Several surgical landmarks should be taken into consideration throughout the approach: foramen ovale and CN V_3_ (identified just posterior to the base of the lateral pterygoid plate); the tensor veli palatini muscle (between the pterygoid plates and just anterior to the Eustachian tube); the cartilaginous Eustachian tube (anterior to the petrous and parapharyngeal ICA and posterior to V_3_).

Aggressive tumors that invade the pterygoid muscles are best managed by an open approach that allows the exenteration of the infratemporal fossa. Intraoperative histological analysis of the surgical margins after any resection is ideal regardless of the surgical approach.

#### Reconstructive technique

After a complete resection is confirmed (negative margins) by intraoperative histological analysis, the reconstruction recreates the arachnoid layer using an inlay graft of collagen matrix (e.g. DuraGen; Integra LifeSciences Corp., Plainsboro, NJ). The nasoseptal flap is then onlaid overlapping the defect widely (allowing for some tissue contraction). Gelatin foam (Gelfoam; Upjohn Co., Kalamazoo, MI) Nasopore (Stryker Corp; Kalamazoo, MI) creates a non-adherent barrier between the flap and the nasal packing. Occasionally, a free fat graft is inserted prior to obliterate a significant dead space such as the sphenoid sinus or clival recess; therefore, enabling an optimal flap positioning and longer reach. If the Hadad-Bassagaisteguy flap is not available, an alternative reconstructive technique should be considered, either a vascularized flap (i.e. temporoparietal fascia flap^17^, occipital galeopericranial flap30., 31., middle turbinate^16^ or inferior turbinate flaps14., 15., and lateral nasal wall flap22., 23.) or free tissue graft. Most commonly, we use a temporoparietal fascia flap inserted through a transpterygoid tunnel.

A temporoparietal fascia flap is harvested via a hemicoronal incision and its passage into the nasal cavity is facilitated by a tunnel that connects the temporal fossa to the infratemporal fossa and eventually to the nasal cavity via the pterygopalatine fossa. This tunnel requires varying degrees of expansion using dissection, bougienage and partial removal of the pterygoid process. The geometry and dimensions of the pterygopalatine fossa, antrum and the soft tissue flap, dictate the dimensions of the tunnel. Finally, the reconstruction is bolstered in place using sponge packing, the balloon of a Foley catheter or both. Silicone splints are used to protect the nasal septum, if needed.

### Endoscopic nasopharyngectomy

#### Indications and limitations

Surgery for primary nasopharyngeal carcinoma has a limited role, as radiotherapy is the mainstay of treatment. Most often, surgery serves to provide tissue samples or to alleviate compressive symptoms. However, current evidence supports that surgery play a significant role in carcinomas of glandular origin and for persistent or recurrent loco-regional disease32., 33., 34., 35.. Surgical resection is one of many acceptable options among a variety of modern re-irradiation techniques including stereotactic, brachytherapy, proton, or intensity modulated therapy36., 37., 38., 39., 40., 41., 42.. Furthermore, surgery plays a primary the role in the treatment of radioresistant tumors such as adenocarcinoma, adenoid cystic carcinoma, mucoepidermoid carcinoma, and sarcomas. Primary resection of these tumors seems advantageous^43^.

Endoscopic endonasal nasopharyngectomy is a challenging operation. Transpterygoid approaches provide exposure to all lateral sub-sites of the nasopharynx, including the infratemporal fossa. However, encasement of the parapharyngeal ICA or extension of the tumor posterior to the ICA (parapharyngeal or petrous segments) is considered a contraindication to an endoscopic resection with curative intent. These extensions require an open approach such as the preauricular or post-auricular subtemporal approaches^44^.

#### Surgical technique (Figure 3)

Intuitively, the extent of the endoscopic endonasal approach and resection matches the tumor location and extensions. A small localized lesion on the central nasopharynx may be resected via a transnasal corridor (including removal of the posterior part of inferior turbinate and a limited infero-posterior septectomy) that allows adequate visualization and facilitates bi-lateral instrumentation. This conduit offers the possibility to resect the posterior and superior nasopharyngeal walls including the bony floor of the sphenoid sinus. Furthermore, the resection can extend antero-superiorly to include the anterior wall and the sphenoid sinus. It may also be extended inferiorly to the level of C_2_ (second cervical vertebra), and posteriorly to reach the periosteum of the clivus.

**Figure 3:**
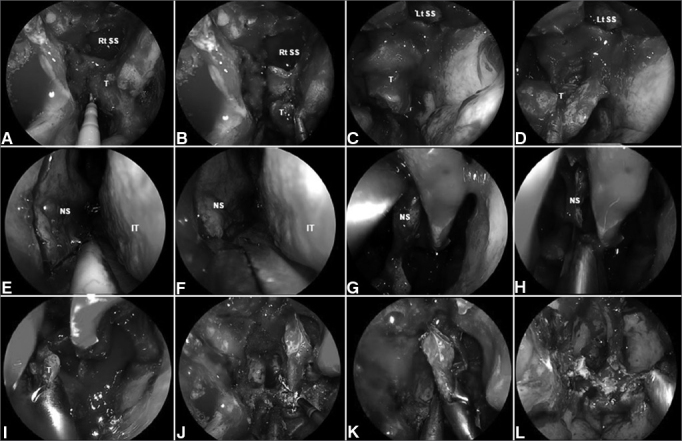
A-D: Intraoperative photograph demonstrating tumor at superior wall of nasopharynx. An incision is made just above the torus tubarius A allowing a subperiosteal dissection of the basisphenoid and clivus; E-K: A posterior septectomy and sphenoidotomies are performed; and the sphenoid sinus intersinus septa are removed; L: Finally, histological analysis is required to confirm the negative surgical margins. Rt. SS: Right sphenoid sinus; Lt. SS: Left sphenoid sinus; NS: Nasal septum; IT: Inferior turbinate.

Unfortunately, the tori tubarius are often involved; thus, the resection of the medial cartilaginous portion of the affected Eustachian tube is required. The posterolateral wall of the nasal cavity is incised just posterior to the inferior turbinate (ascending process of the palatine bone) and a mucoperiosteal dissection follows to expose the medial aspect of the medial pterygoid. This subperiosteal plane may be followed to elevate the soft tissues covering the roof of the nasopharynx and facilitating the transection of the torus.

Lateral involvement of the fossa of Rosenmüller requires a transpterygoid approach including a medial maxillectomy and transposition of the soft tissue contents of the medial pterygopalatine fossa to expose the entire height of the pterygoid process, and to allow the removal of the medial pterygoid plate and its base (to the level of foramen rotundum). Any anatomical variation requiring a modification of this template can be identified and planned for using the preoperative imaging.

Whenever possible the lateral pterygoid plate is preserved as it serves as a landmark to mark the position of V_3_ (just posterior to its base) and the parapharyngeal ICA (posterior to V_3_); therefore, these three structures align on the sagittal plane. Tumor extension to the pterygopalatine fossa and infratemporal fossa requires expansion of the transpterygoid corridor (discussed above). The dimensions of this corridor depend on the geometry and pneumatization of the maxillary sinus and nasal cavities (inherent), and the extension of the tumor (to be followed by the surgeon). If the endoscopic medial maxillectomy only affords a limited exposure of the infratemporal fossa, we can extend it anteriorly (endoscopic Denker's approach) as previously discussed.

After complete exposure of the posterior wall of maxillary sinus, the pterygopalatine soft tissue contents are meticulously dissected and the bone around the vidian canal and foramen rotundum is drilled out in an anterior to posterior fashion. The mandibular branch of the trigeminal nerve (CN V_3_) is identified anterior to the petrous ICA and under CN V_2_. Following the identification of the lacerum segment of the ICA's, the bone covering its petrous (horizontal) segment is removed laterally. When distal control of the ICA is necessary, the bony canal over the paraclival carotid (vertical segment) is also removed. Dissection of the tumor along the ICA is performed with great care, confirming the position and trajectory of the vessel with both the intraoperative navigation and acoustic Doppler sonography. The medial Eustachian tube is transected and removed to expose the entire fossa of Rosenmüller and the tumor within. The tumor is removed en bloc or in sequential layers according to its relationship to critical neurovascular structures.

#### Reconstructive Technique

Reconstruction of the surgical defect, including coverage of the middle cranial and/or posterior cranial fossae and exposed ICAs, must be carefully planned including the possibility of needing alternative options. Harvesting of a contralateral Hadad-Bassagaisteguy flap must be completed at the beginning of the surgery and stored in its ipsilateral maxillary antrum, sphenoid sinus or against the lateral nasal wall. The Caicedo reverse flap is raised and mobilized to cover the septal cartilage donor site, left bare after harvesting the Hadad-Bassagaisteguy flap.

Following histological confirmation of tumor-free surgical margins, the defect is reconstructed in similar fashion to that previously described using a collagen matrix graft and a vascularized flap. However, if the extent of the defect is larger than the potential coverage of the Hadad-Bassagaisteguy flap, or this flap is not available, reconstruction proceeds with a temporoparietal fascia flap. The latter can be introduced into the nose via a transpterygoid tunnel to cover exposed dura and/or ICA17., 45..

### Endoscopic transclival resection

#### Indications and limitations

Malignant tumors invading or originating from the clivus are rare, but usually aggressive and locally destructive tumors (i.e. chordomas, chondrosarcomas). Radical resection and postoperative radiotherapy are the standard treatment modalities. However, surgical approaches to the clivus are challenging due to its intricate and deep-seated anatomy with abundant adjacent critical neurovascular structures.

Endoscopic endonasal transclival approaches provide the most direct route to the median, ventral posterior fossa without retraction of brain. Rostrally, they can access the retrosellar region, extending to the interpeduncular fossa (via a pituitary transposition)^46^; whereas caudally, the can effectively extend to the level of C_2_^5^. Lateral exposure can be extended incorporating a transpterygoid corridor. If an endonasal access does not provide adequate lateral access, the tumor resection may be staged, using an external lateral approach to address any residual tumor.

In general, the guiding principle for the resection is to avoid crossing nerves; thus, an EEA is best to access tumors ventral to the cranial nerves. A significant dorsal extension requires an alternative approach (as an adjunct). Other limitations include significant lateral extension to the cavernous sinus or the paraclival carotid artery or marked posterolateral extension encasing the basilar and/or vertebral arteries or invading brain stem^47^.

#### Surgical technique (Figure 4)

The sinonasal corridor is created as previously described to combine the posterior nasal cavities into single conduit. This allows for exposure of the sella, the upper third of the clivus, and the paraclival ICA. Lateral widening of the sphenoidotomy expose the lateral recesses (bilateral). Exposure of the middle and lower thirds of the clivus is completed with the removal of the mucosa of the nasopharynx and the underlying basopharyngeal fascia and the insertion of the longus capitis muscles. Subperiosteal dissection should keep between the Eustachian tubes, particularly when using the electrocautery, as the ICAs run just lateral and posterior to the Eustachian tubes46., 48., 49.. Occasionally, the ICAs may be ectatic (more common in the elderly) and at a median position between the Eustachian tubes. This should be identified in the preoperative imaging.

**Figure 4:**
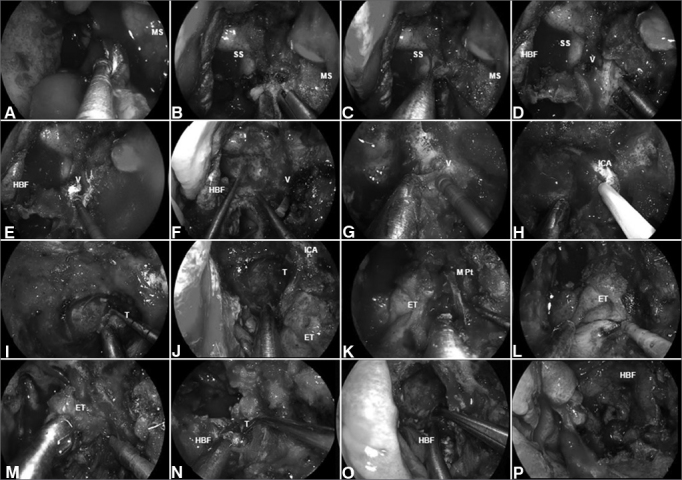
A: Malignant clival tumor extending laterally beyond the paraclival ICA; therefore, requiring endoscopic transpterygoid. After a total ethmoidectomy and bilateral sphenoidotomies, a medial maxillectomy is completed to access the pterygoid process; B: The sphenopalatine artery is coagulated before removing the posterior wall of maxillary sinus; C: The greater palatine canal opened and the descending palatine artery and the greater palatine nerve are freed. Sequentially, the vidian nerve is transected to allow the lateral displacement of the soft tissue contents of the pterygopalatine fossa; D: After adequate exposure of anterior aspect of the pterygoid process; E-F: The floor of the sphenoid sinus is drilled flush with the level of the clival recess; G-H: Subsequently, the vidian canal is dissected and the ICA position is confirmed with nasal acoustic Doppler sonography; I-J: Removal bony of the pterygoid base enhances the exposure for tumor removal from the clivus; K-M: The medial pterygoid plate and Eustachian tube are removed; N-O: Allowing a total resection of the clival tumor with adequate margins; P: Finally, the HBF is placed to cover the defect and ICA. MS: Maxillary sinus; SS: Sphenoid sinus; HBF: Hadad-Bassagaisteguy nasoseptal flap; V: Vidian nerve; ICA: Internal carotid artery; M. Pt: Medial pterygoid artery; T: Tumor; ET: Eustachian tube.

Removal of the clivus is performed using a high-speed drill with a 3–4 mm hybrid or coarse diamond burr. The venous plexus posterior to the clivus (basilar plexus) is quite rich and bleeds significantly; thus, requiring frequent plugging with hemostatic paste, microfibrillar cellulose, or gelatin sponge.

Tumor extending lateral or posterior to the plane of the paraclival or petrous ICA mandate surgical control of these segments. The anterior genu between the horizontal petrous segment and the vertical paraclival ICA (at the level of the foramen lacerum) is best identified by the vidian artery and nerve running in the vidian canal50., 51..

A transpterygoid approach is performed following the previously described technique to expose the Eustachian tube. The ICA anterior genu is by drilling out the exposed “medial pterygoid wedge”. Once the ICA is isolated, it can be mobilized lateral and cephalad, providing access to tumor extending posterior to it. An endoscopic Denker's approach can be added for extended lateral access, facilitating removal of the medial and lateral pterygoid plates and adjacent soft tissue. This is most commonly required to gain control of the parapharyngeal ICA, using the Eustachian tube and V_3_ as superficial landmarks. In addition to its lateral access, this approach provides a corridor that facilitate the removal of tumor extending to the foramen magnum, hypoglossal canal, medial occipital condyle, and jugular foramen. Therefore, using a combination of approaches one can achieve complete control and mobilization of the ICA (parapharyngeal, horizontal petrous, anterior genu, and vertical paraclival segments), and allow access to tumor to the ICA from both medial and inferior trajectories.

Inn addition to the ICA, one must remain cognizant of the course of any involved cranial nerves, especially the CN VI, which can be injured at every stage of the approach and resection. Bone drilling or tumor resection adjacent or posterior to the superior paraclival ICA, can damage CN VI as it exits Dorello's canal and courses toward the cavernous sinus. Another critical anatomic landmark to avoid injury to CN VI is to realize its relationship to the vertebrobasilar junction^52^. CN VI arises at the level of the vertebrobasilar junction and then runs obliquely in the prepontine cistern to Dorello's canal. The best way to avoid injury at this area is to open the dura (when needed) below the anticipated location of the vertebrobasilar junction at approximately the level of the basion (roughly corresponds to the floor of the sphenoid sinus). We prefer to use a bimanual “2-suction” technique for internal debulking of soft or gelatinous tumors such as chordomas or chondrosarcomas^53^. Intradural resection is limited by adherence of the tumor capsule to perforator vessels, cranial nerves, and brainstem. Therefore, the presence of subarachnoid planes becomes a limit of the surgery regardless of the surgical approach. To that end, tumor should never be blindly pulled. If needed, the exposure should be extended and dissection should be performed only under direct visualization^53^.

#### Reconstruction technique

Reconstruction of the resulting defect most commonly uses an intradural collagen matrix graft (or fascia lata) and the Hadad-Bassagaisteguy flap as previously described. A temporoparietal fascia flap is recommended if the nasoseptal flap is not available. A long bony tunnel, created during the transclival drilling, may require obliteration with free abdominal fat to allow a full coverage with the flap. A temporary packing (3–5 days) bolsters the reconstruction. Gelatin foam (e.g. Gelfoam; Upjohn Co., Kalamazoo, MI) or Nasopore (Stryker Corp; Kalamazoo, MI) applied over the flap creates a non-adherent barrier between the flap and the nasal packing.

## POSTOPERATIVE CARE

Upon completion of the surgery, the patient is transferred to a monitored unit that facilitates early detection of complications and provide attention to fluid and metabolic disturbances that can occur during the early postoperative period; i.e. two common postoperative disturbances include diabetes insipidus (DI) and the syndrome of inappropriate secretion of antidiuretic hormone (SIADH). In addition, the physician should be alert to others major complications that can occur after skull base surgery, especially significant intracranial hemorrhage or tension pneumocephalus. These early complications may be identified with a postoperative non-contrasted CT scan immediately after surgery (on a stable patient). We also advocate a contrasted MRI within the first 24 hours after surgery to corroborate the completion of the resection. In addition, the contrast uptake may provide an idea of the vascularity of the reconstructive flap as well as the adequacy of its positioning.

Indwelling lumbar spinal drainage is rarely indicated. We reserve its use for patients who are considered at high risk for increased intra-ventricular pressure and for those with an intraoperative high-flow leak (defined as opening of the third ventricle or at least 2 cisterns).

## DISCUSSION

Advances in endoscopic endonasal surgery have enabled the extirpation of sinonasal carcinoma. Endoscopic resection techniques include a step-by-step dissection based on anatomical principles, constant assessment of tumor boundaries followed by, a piecemeal or layered removal and intraoperative histological analysis of surgical margins. Recent publications support the oncologic adequacy the endoscopic endonasal approaches even when the tumor is not removed en bloc. Conversely, one should not assume that the use of an open approach assures better margins. Patel et al.^54^, reported a multinational, multi-institutional study of anterior craniofacial resections in which close to a third of the patients (32%) had close or positive margins. This can be explained by the the complex relationship of these tumors to the critical anatomy of the skull base, which in turn drives the surgeon to remove the tumor with minimal margins in order to preserve a vital structure (in these cases quality of life and safety supersedes the standard oncological wide margins).

One difficulty when analyzing the data regarding endoscopic surgery is the different terminology regarding what constitutes an endoscopic endonasal or endoscopic-assisted surgery. We will only discuss those studies in which the use of the endoscope was clearly defined as the only visualization tool during an endonasal approach and resection (i.e. pure endoscopic endonasal approach reconstructed with free tissue grafts, or endonasal or regional flaps) and those in which a craniotomy with or without a sub-basal approach was combined with an endonasal endoscopic approach to avoid facial incisions (i.e. endoscopic-assisted or cranioendoscopic approach). Those case series in which the endoscope was used to inspect the surgical field through standard open approaches will not be discussed.

Higgins et al.^55^ conducted a systematic review comparing the oncologic outcomes after endoscopic endonasal approaches and those of traditional open approaches. Both techniques yielded similar outcomes (difference not statistically significant), including 5-year overall survival (87.4 ± 5.3% in the endoscopic group and 76.8 ± 8.3% for open approach; *p* = 0.351), disease-specific survival (94.7 ± 3.7% in the endoscopic group and 87.7 ± 6.7% for open approach; *p* = 0.258), and locoregional control rate (89.5 ± 5.0% in the endoscopic group and 77.2 ± 10.4% for open approach; *p* = 0.251).

Others, however, have reported differences in outcomes. Eloy et al.^56^ published their experience after open and endoscopic endonasal anterior skull base resection of sinonasal malignancies reporting no significant statistical difference in overall survival (endoscopic group = 94.4% vs. open craniofacial group = 83.3%). Nonetheless, the difference in local recurrence rate did approach statistical significance (5.6% for the endoscopic group and 29.2% for open craniofacial group; *p* < 0.05)). Kim et al.^57^ studied 40 sinonasal malignancies (19 esthesioneuroblastoma, 7 squamous cell carcinoma, 3 malignant melanoma, and 11 others) invading the anterior skull base. Their outcomes regarding local recurrence were similar to those of Eloy et al.^56^, demonstrating that 11% of the patients undergoing an endoscopic approach developed a recurrence; whereas 55% of those undergoing an open approach recurred. One should recognize a strong patient selection bias in both studies, as a larger proportion of tumors with advanced stages was treated by the open approach. In addition, Kim et al.^57^ inappropriately mixed benign and malignant lesions in their analysis.

Suh et al.^58^ assessed the outcomes of endoscopic and endoscopic-assisted resection (i.e. craniotomy or sub-basal approach combined with endoscopic endonasal approach) in a case series including heterogeneous histologies including sarcomas (9 patients), squamous cell carcinomas (8 patients), adenocarcinomas (8 patients), and melanomas (7 patients). The 3-year disease-free survival rate was 86.8% for the endoscopic endonasal group and 67.7% for the endoscopic-assisted open group with a mean follow up time of 3.58 years. Overall local tumor recurrence rate was 16% and disease-specific mortality was 8%. These outcomes are similar to those of a large retrospective series published by Nicolai et al.^59^, who reported a 5-year disease-specific survival of 91.4 ± 3.9% for an endoscopic-endonasal cohort, which was superior to the 5-year disease-specific survival for a cranioendoscopic (i.e. endoscopic-assisted) group, which was 58.8 ± 8.6%. Recurrent disease was observed in 23.6% for the entire patient cohort (43/182), and included 19% (25/133 patients) of the endoscopic group and 37% (18/49 patients) of the cranioendoscopic group; therefore, suggesting that endonasal endoscopic and endoscopic-assisted approaches for the management of adequately selected sinonasal malignancies are a reasonable alternative to traditional open approaches. However, some remaining controversies require further study with stratification of survival data by histology and stage, as well as longer follow up^60^.

Similarly, in a meta-analysis by Devaiah & Andreoli^61^ endoscopic approaches provided superior survival rates (*p* = 0.0019), even when stratifying for publication year (*p* = 0.0018). Median follow-up was 51 months for patients undergoing a traditional craniofacial resection and 54.5 months for those in the endoscopic group. These apparently “superior” outcomes after endoscopic endonasal resection should be carefully interpreted, considering confounding factors such as follow-up times, stage, invasiveness, and histopathology that undoubtedly influenced these results.

Nonetheless, a study of esthesioneuroblastoma by Folbe et al.^62^ suggests that Kadish C and D tumors can be effectively treated using an endoscopic endonasal approach followed by postoperative radiation therapy. This series included patients with esthesioneuroblastomas that were staged as Kadish A (10.5%), B (58.9%), C (26.3%), and D (5.3%); with an average follow-up (primarily treated cases) of 45.2 months (11–152 months). At the time of publication all patients were alive and free of disease. Similar findings have been recently reported study by Gallia et al.^63^, who presented their experience resecting esthesioneuroblastomas via endoscopic endonasal approaches (modified Kadish stage A (18.2%); stage B (18.2%); stage C (45.5%); and stage D (18.2%). All patients underwent a complete resection obtaining negative margins intraoperatively; and, at an average follow-up of over 28 months, all patients were alive and free of disease. Of note, the mean period to develop a recurrence is 6 years^64^; therefore, long-term follow-up seems critical.

In addition, it is important to recognize that the management of sinonasal tumors with anterior skull base involvement remains multimodal. Despite some remaining controversy, most patients with sinonasal malignancies are treated with a combination of surgery and radiation therapy. Outcomes after treatment including neoadjuvant chemotherapy followed by surgery and postoperative radiation therapy or primary radiation therapy were evaluated by Song et al.^65^, who retrospectively studied 35 patients including 7 who were treated with chemoradiotherapy, 12 who underwent a traditional craniofacial resection, 11 an endoscopic-assisted resection, and 5 patients who had a pure transnasal endoscopic resection. At an mean follow-up period of 64.9 months the 5-year disease free survival rate was 35.7% for the nonsurgical treatment group, 41.7% for the traditional craniofacial resection, 80.8% for the endoscopic-assisted resection, and 100% for the purely transnasal endoscopic resection (*p* = 0.01) groups. Overall local recurrence was observed in 23% of the patients, but specifically in 33% of patients after traditional craniofacial resection, 9% after endoscopic-assisted resection, and 14% after nonsurgical treatment. No recurrences were observed in patients who underwent a purely endoscopic transnasal resection.

Endoscopic endonasal surgery is an important modality of treatment for tumors involving the posterior cranial base including nasopharynx, clivus and craniovertebral junction (i.e. nasopharyngectomy, transclival resection). Endoscopic endonasal nasopharyngectomy plays an important role in residual or recurrent loco-regional tumor, and malignancies of glandular and mesenchymal origin. In addition, surgical resection followed by adjunctive radiation therapy remains the mainstay treatment for chordomas and chondrosarcomas.

Similar to the resection of the anterior skull base, a nasopharyngectomy can be performed using various open approaches (i.e. maxillary swing, LeFort I, preauricular temporal-subtemporal) or endoscopic endonasal approaches. Their oncologic outcomes seem similar assuming an adequate selection of patients. Chan et al.^66^ reported the largest retrospective review of outcomes in 268 patients after salvage nasopharyngectomy via maxillary swing. These authors reported that they were able to obtain clear resection margins in 79.1% of patients, obtaining a 5-year actuarial local tumor control and overall survival was 74% and 62.1%, respectively (median follow up of 52 months). Conversely, early reports analyzing the outcomes of endoscopic endonasal nasopharyngectomy demonstrated a 2 year disease free survival rates of 57.6–86.3%, and 2 year overall survival rates of 59.4–100%67., 68., 69.. These series suggested that the endoscopic endonasal approach was beneficial for patients with early stage carcinomas. Nonetheless, in a series of extended endoscopic transpterygoid nasopharyngectomy for advanced primary and recurrent malignancies published by Al-Sheibani et al.^43^ early outcomes demonstrated an overall survival of 45% (9/20) and a local control of 65% (13/20). These outcomes have been reproduced by Castelnuovo et al.^70^, who recently reported their experience with endoscopic nasopharyngeal resection, including patients with advanced stage. Their study included 36 consecutive patients with primary (9 patients) and locally recurrent (27 patients) nasopharyngeal carcinomas, including patients with stage I (44.4%), stage II (8.4%), stage III (41.6%), and stage IVA (5.6%) and a mean follow up period of 38 months. Overall oncologic outcomes at 5 years survival rate, disease specific, and disease free survival were 75.1 ± 9.13%, 80.9 ± 7.79%, and 58.1 ± 14.8%, respectively. However, the reproducibility and efficacy of endoscopic transpterygoid nasopharyngectomy require validation with larger case series and longer follow up.

Recently, the use of the Da Vinci robot (Intuitive Surgical, Sunnyvale, California) has been suggested as an adjunctive tool to facilitate of skull base surgery. It provides three-dimensional (3D) visualization, affords 2 to 3-handed surgery (robotic arms and co-surgeon/assistant's), and has the advantage of wristed instruments that improve the ability to manipulate the tissue (e.g. dural resection or repair)71., 72.. The potential for robotic skull base surgery has been evaluated in various cadaveric models73., 74., 75., 76., 77., 78.. Hanna et al.^79^ described a bilateral transantral approach to facilitate accessing the anterior and central skull base. Dallan et al.^80^ reported a feasibility study illustrating the advantages of a transnasal-transcervical/transoral robotic surgery. These authors studied the feasibility of using the robotic camera through the nasal cavity and inserting transcervical/transoral trocars as corridors for the Endowrist robotic arms, as previously suggested by O'Malley & Weinstein^74^, as well as Lee et al.^78^. They concluded that this combined technique avoided the need for a palatal transection and that it seemed adequate for the resection of small tumors. Various approaches using combinations of camera corridors and Endowrist instrument ports were assessed by Ozer et al.^71^ A transoral camera (30°) and instruments provided good control of the posterior and lateral nasopharynx; but access to the roof of the nasopharynx was inadequate. A transnasal camera (0°) and transoral instruments provided great visualization but instrumentation was limited and cumbersome. Overall, the transpalatal approach (hard palate) offered the best combination of visualization and ease of instrumentation. Adjunctive transcervical ports for the Endowrist instruments, however, provided superior range and ease of instrumentation with all the camera corridors.

It should be noted, however, that the robot does not have a drill or suction device. Therefore, the endoscopic endonasal techniques complement the robotic techniques, allowing drilling of the skull base and controlling areas not fully accessed by the robotic approach. Yin et al.^81^ were the first to report the clinical use of an endoscopic endonasal approach combined with a transoral robotic resection of a small recurrence at the superior aspect of the nasopharynx, Subsequently, a combined technique, using transoral robotic surgery and an endoscopic transpterygoid approach, to remove extensive malignant tumors of the posterior skull base, nasopharynx and ITF was developed and applied by Carrau et al.^82^

Tumors in the clival region present challenges to the surgical team due to their encroaching nature, proximity to critical neurovascular bundles and large volume at presentation. Chordomas and chondrosarcomas are the most common clival malignant tumors and their therapy options include surgery and various forms of radiotherapy. Regarding the philosophy and technical approaches for the surgical resection of the clival region they range from the conventional open to minimally invasive endoscopic endonasal approaches. Their oncologic outcomes have been demonstrated. Gay et al.^83^ described their experience with a variety of open approaches including subtemporal, transzygomatic, transcavernous, and transpetrous apex; subtemporal and infratemporal; extended frontal; and extreme lateral transcondylar approaches in 60 patients. These approaches allowed a total or near total resection of 67% and a recurrence-free survival rate of 80% at 3 years and 76% at 5 years. Another large series by Di Maio et al.^84^, described their experience with open surgical resection of chordomas in 90 patients, yielding a 5-year overall and recurrence free survival of 74 ± 6% and 56 ± 8%, respectively. These results were similar to studies reporting clinical series of cranial base chordoma resected by open approaches that reported a complete resection rate that ranged from 23.1% to 71.6%. 5-years progression free survival ranged from 15% to 64%, and 5-years overall survival ranged from 65% to 82.4%85., 86., 87., 88., 89..

Similarly, outcomes of endoscopic transclival resection have been reported by several studies. Fraser et al.^90^ reported that > 95% resection was achieved in 7 of 8 operations (5 of 6 patients), and 80% of the tumors was resected in the remaining patient. Holzmann et al.^91^ found that endoscopic endonasal approaches yield radical or near total removal of clival chordoma in 12/13 patients. Stippler et al.^53^ presented the treatment of the 12 newly diagnosed chordomas including 8 total resections (66.7%), 2 near total resections (16.7%), and 2 subtotal resections (16.7%). Endoscopic surgery of 8 recurrent chordomas included 1 gross total resection (12.5%), 2 near total resections (25.0%), and 5 subtotal resections (62.5%). Two (10%) of these patients suffered a recurrence, and 5 patients (25%) had progression of the disease during a mean follow-up period of 13 months (range, 1–45 months). Saito et al.^47^ reported that a gross total removal was achieved in 3 of 6 patients. An incomplete resection (residual tumors) was mainly due to chordoma in the epidural and subdural spaces. A recent study of the endoscopic resection of cranial base chordomas, published by Koutourousiou et al.^92^, reported an overall rate of gross total of 66.7% (82.9% in primary cases and 44% in previously treated patients). Important limitations to achieve a gross total resection included: a tumor volume greater than 20 cm^3^ (*p* = 0.042), tumor location in the lower clivus with lateral extension (*p* = 0.022), and previously treated disease (*p* = 0.002).

Endoscopic transclival resection of chondrosarcomas is often reported in combination with chordoma; thus, their outcomes seem similar. However, their biological behavior differs significantly. Chordomas tend to invade the dura more frequently and have a greater propensity for local recurrence. Chondrosarcomas of the skull base are typically slow growing lesions that most commonly present at the petroclival junction. Their behavior correlated with their histological grade. Frank et al.^93^ reported the extent of extirpation of chordomas and chondrosarcomas in 11 patients. Patient follow-up periods ranged from 15 to 69 months (mean, 27 months). Three patients died of chordoma progression at 20, 14, and 10 months, respectively, after endoscopic treatment. One patient experienced two recurrences. They encountered no recurrence in 2 patients with chondrosarcoma (both had undergone a radical resection). Another study reported by Zhang et al.^94^ that they achieved total resection was in 6 patients of chordoma and 1 case of chondrosarcoma, and a subtotal resection in a patient with chordoma and another patient with chondrosarcoma. At their last follow-up, 7 patients had no evidence of disease and 1 was alive with disease (a patient with chordoma suffered a recurrence 5 months after a subtotal removal).

Chordomas and chondrosarcomas are rare; thus, accruing a significant number of cases is difficult for a single institution. Furthermore, outcomes after endoscopic approaches cannot be directly compared with those of the open approaches, as there is a strong selection bias. Nonetheless, the outcomes suggest that the endoscopic transclival approach is feasible, yields an appropriate extent of resection and acceptable oncologic outcomes. Furthermore, one should consider that advanced chordomas and chondrosarcomas often require multi-stage surgery using multiple approaches. Their ultimate outcome is also dependent on the adequacy of adjunctive radiation therapy.

Rate and severity of surgical complications is a critical consideration when choosing among possible surgical approaches. The skull base anatomy is complex and comprises multiple critical neurovascular structures. One of the great surgical challenges is the avoidance and management of catastrophic bleeding (i.e. internal carotid arteries or other major vessel). Prevention of injury to the carotid artery is based on a proper understanding of skull base anatomy from an endoscopic perspective, thorough reviewing of the preoperative imaging, use of adjunctive tools such as image guidance and acoustic Doppler sonography, and cautious dissection. A fundamental tenet is that any injury to the ICA must be controlled while maintaining cerebral perfusion. Therefore, neurophysiologic monitoring is helpful as it reflects cerebral perfusion. Digital compression of the cervical carotid may diminish its flow, although the effectiveness of this maneuver is variable and its logistics not as simple as it appears (the two operating surgeons will have their hands occupied controlling the bleeding and is difficult to accommodate a third person around the head of the patient). Management by the anesthesiologist is fundamental and should include adequate and prompt resuscitation with fluids and blood products. Use of hypotensive anesthesia as an attempt to control the bleeding is contraindicated since this results in cerebral hypoperfusion.

A key measure that may appear counterintuitive is the administration of heparin to avoid embolic phenomena. Ultimately, a two-surgeons-four-hands technique with dynamic handling of the endoscope to preserve an adequate view of the surgical field and the use of two suctions offers the best opportunity to identify and control the site of bleeding. Initially the bleeding is directed into the suction tips to maintain visualization while focal pressure is applied with a cottonoid. The endoscope is advanced through the nostril with the least blood flow and visualization is maintained by cleaning the lens with saline solution (manually or by a lens cleansing device)95., 96..

Concomitantly, an assistant harvests muscle from the thigh (preferred) or abdomen. Some have advocated harvesting muscle from the temporal area or even the tongue; however, this is difficult to do around the surgeons that are controlling the bleeding, the volume of muscle may be inadequate; and, in the case of the tongue, it will be contaminated with oral flora and may produce further permanent deficits of speech. The muscle is then directly applied over the injury to elicit hemostasis95., 96.. It should be noted that a muscle patch may require 40–45 min to seal the vessel. Once hemostasis and resuscitation are successful and the patient vital signs and neurophysiologic monitoring are stable, additional packing could be placed to hold the muscle in place and the patient may be transported to an angiography suite for definitive management. Endovascular sacrifice of the ICA is the most commonly used alternative; however, it is best performed after assessment of collateral blood flow with a balloon occlusion test (estimates the risk of ischemic stroke and/or the need for bypass).

Using of a covered stent^97^ may preserve the patency of the ICA; however, they are not available for intracranial use in all institutions. In addition, deployment of a covered stent into the cavernous sinus segment of ICA is technically challenging and requires at least 6 weeks of antiplatelet therapy. The latter is an important consideration if the tumor needs to be removed or debulked urgently. A follow-up angiography is also recommended after any intraoperative vascular injury. These patients are at risk for a delayed pseudoaneurysm and rupture that can present weeks to years after the event^98^.

Gardner et al.^99^ reviewed the incidence of ICA injury in 2015 endoscopic skull base surgeries. The authors reported an incidence of 0.3% (7 patients); however, to include all patients in the denominator for this calculation is incorrect. Patients with pathologies of the orbit and anterior cranial fossa should have no risk of injuring the ICA; thus, the incidence is underestimated for patients with pathologies adjacent to any of the segments of the ICA. It is interesting to note that most of their injuries (5 of 7 patients) involved the left ICA; that the most common diagnosis associated with an ICA injury was that of a chondroid neoplasm (i.e. chordoma, chondrosarcoma; 3 of 7 patients); and that the transclival and transpterygoid approaches were associated with a higher incidence of injury. The paraclival ICA segment was the most commonly injured site (5 of 7 patients. A recent verbal communication by the same group reported on the use of various maneuvers to stop the bleeding including bipolar electro-cauterization of the vessel to weld the tear shut or to induce thrombosis of the vessel, direct compression, compressive packing, suture repair, reconstruction using aneurysm clips, and circumferential ligation or clipping of the vessel.

They also advocated attempting the preservation of lumen by reconstructing the vessel with suture repair, aneurysm clips, or using Sundt-Keyes clips. In our experience, all these maneuvers require further exposure of the vessel and are associated with significant blood loss with a subsequent array of various systemic complications. In addition, it is important is to note that compressive sinonasal packing is not an option if the dura is opened, as blood will track into the subdural space. Furthermore, this group also reported a catastrophic complication arising from the injection of hemostatic paste into the injury; thus, leading to catastrophic embolic phenomena. Skull base surgeons should be aware of this possibility and consider ill advised to use this technique unless the surgeon is sure that the ICA or other major artery is not injured. In our experience, the most effective control of an arterial injury is by direct control of the injury site using cottonoids followed by the direct application of crushed muscle (harvested from the abdomen or thigh). Valentine et al. have corroborated the effectiveness of this maneuver in an elegant experimental model95., 96..

Despite its dramatic impact a vascular injury is not the most common major complication associated with endoscopic endonasal skull base surgery. This undesirable title belongs to postoperative CSF leak^9^. Its incidence, however, has decreased significantly (< 5%) after the adoption of vascularized tissue flaps.

Kassam et al.^9^ reported other complications including transient neurological deficits (2.5%), permanent neurological deficits (1.8%), intracranial infection (1.6%), systemic complications (2.1%) and death (0.9%).^9^ In addition to the potential risk for vascular complications, extended endoscopic approaches to the infrapetrous region or Meckel's cave present a relatively high risk for other complications including injury to CN V or VI. Attentive dissection and the use of adjunctive electrophysiologic monitoring help to diminish the risk of injury to the cranial nerves. Reduction of lacrimation (i.e. dry eye) is of concern in the postoperative period (especially in patients with CN V_1_ dysfunction and/or facial paralysis, who cannot protect the cornea adequately), and may occur due to injury of the sphenopalatine ganglion or vidian nerve. Trismus is common after dissection of the pterygoid muscles. Analgesics and stretching exercises should be started early in the postoperative period to avoid permanent and progressive scarring of the muscles that could lead to severe limitation of the oral opening.

Pant et al.^100^ reported sinonasal complications that included nasal synechiae (9%), alar sill burn/abrasions (5%), maxillary nerve hypoesthesia (2%), palatal hypesthesia (7%), incisor hypesthesia (11%), serous otitis media (2%), taste disturbance (7%) and malodor (19%). These sinonasal complications were frequently temporary.

Comparing their experience with endoscopic and open resections of the anterior skull base, Eloy et al.^56^ reported that the incidence of significant complications was greater in the group undergoing an open approach. In the group of patients undergoing endoscopic resect-ion, they encountered three CSF leaks, two nose-bleeds, one altered mental status, and one nasal stenosis. Conversely, in the group undergoing an open approach three patients developed a postoperative CSF leak, three patients developed diplopia, one developed an epidural hematoma, one suffered a conjunctival tear with ipsilateral blindness, one had an ectropion, one suffered a wound dehiscence, and one developed sepsis. An assessment of postoperative radiological findings after anterior craniofacial resection demonstrated that more than a third of the patients suffer significant edema or contusion and that 75% eventually develop encephalomalacia^101^. Furthermore, Ganly et al.102., 103. surveyed the morbidity and mortality of craniofacial resections in a collaborative international study, subsequently reporting a complication rate of 36% and a mortality of *5%*, and that both rates are significantly higher in the elderly population.

Quality of life (QOL) is of vital interest for patients seeking therapeutic intervention. Several authors have demonstrated the quality of life benefits of endoscopic endonasal approaches. When compared with open approaches, endoscopic approaches cause less postoperative pain and discomfort, and are associated to shorter operative time and length of hospital stay^104^. Eloy et al.^56^ showed a significant difference in the length of hospital stay (mean of 3.8 days for the endoscopic group and 8.1 days for the open group). Cavel et al.^105^ evaluated quality of life using an anterior skull base surgery questionnaire (ASBS-Q). Following endoscopic endonasal resection, 30 of his 41 patients (75%) reported improvement or no change in overall quality of life. Another study by McCoul et al.^106^, assessed the impact of endoscopic skull base surgery on both site-specific quality of life, using the anterior skull base questionnaire (ASBQ), and quality of life related to the sinonasal tract, using the Sino-Nasal Outcome Test (SNOT-22). At 3 and 6 weeks after surgery, the quality of life by ASBQ was stable showing neither a significant decline nor improvement.

However, at 12 weeks postoperatively there was significant improvement of quality of life, which was maintained at 6 months after surgery (*p* < 0.05). Preoperative quality of life was significantly worse in patients who needed revision surgery and it improved significantly in the postoperative period in those patients who underwent gross-total resection (*p* < 0.05). Scores on the SNOT-22 worsened at 3 weeks postoperatively and returned to baseline thereafter. Of note, the use of a nasoseptal flap or a presence of a denuded donor site did not contribute to a decreased quality of life. Bedrosian et al.^107^ showed a reduction in smell and taste at 6 weeks following endoscopic endonasal pituitary surgery using a disease-specific quality of life questionnaire. Smell and taste progressively returned to baseline at one year. Alobid et al.^108^, using the Barcelona Smell Test (BAST-24), reported similar results, finding that patients experienced a reduction in their sense of smell, which was more pronounced in those patients who required expanded approaches. In addition, others have studied the influence of using a nasoseptal flap over the incidence of postoperative hyposmia and anosmia reporting conflicting results100., 109., 110.. This suggests that many of these quality of life issues require better definition with larger scale studies. Therefore, these studies suggest that endoscopic skull base surgery is a valuable approach for the surgical management of anterior skull base pathology, improving site-specific quality of life. It is important to recognize that the discussion regarding some of the sinonasal quality of life issues, such as crusting, anosmia, and nasal obstruction occurring after endoscopic endonasal surgery for malignancies needs to consider that similar effects accompany the open approaches. Oncologic surgery requires ample exposure and a complete ablation with wide margins, with inherent destructive consequences; thus, these needs trumps mucosal preservation.

Moreover, open approaches seem to be associated with greater risk of wound seeding than endoscopic endonasal approaches. This is an important consideration as it frequently portends a poor prognosis. Moore et al.^111^ estimated that the risk of tumor incisional recurrence at 1 year was 3% for all sinonasal malignancies and 7% for patients with squamous cell carcinoma. This numbers are significant.

Any surgical approach has intrinsic limitations that should be considered. Open techniques have difficulty visualizing anatomical structures at deeper and out of line-of-sight sites, including the orbital apex, frontal recess, and sphenoid sinus, among many others^112^. Rod-lens rigid endoscopy provides magnification, distal illumination and visualization (of the surgical target) and offers the possibility of angled lenses (0, 30, 45, 70 degrees) to look around corners and examine structures at the deeper levels of the surgical field. Conversely, the endoscopic approach only offers a monocular view (lacks depth perception); thus, neurosurgeons accustomed to binocular visualization need to develop compensatory maneuvers. Novel technologies such as three-dimensional endoscopes (provide stereoscopic vision) and other improvements in endoscopic optics, high definition cameras and monitors and will eventually eliminate this obstacle. Advantages and caveats of three-dimensional endoscopy have been proposed in various studies113., 114., 115., 116., 117. and its clinical feasibility has also been demonstrated118., 119.. Of note, Felisati et al.^120^ reported that surgeons encounter some early difficulties that required adaptation (strain, dizziness, difficulties in anatomical orientation, and difficulties in performing the surgical movements).

Endoscopic approaches seem beneficial and advantageous in adequately selected patients; however, they require specialized technical expertise and significant experience. Large and highly invasive tumors, such as those with anterior extension to the cranial convexity, lateral extension over the orbital roofs and those tumors that mandate a total maxillectomy; an orbital exenteration or the sacrifice of the facial skin should be operated using an open approach (an endoscopic approach may be adjunctive). Additionally, an unexpected tumor extension during an endoscopic approach may require conversion to an open procedure; thus, a skull base surgery team should be able to perform endoscopic, endoscopic assisted and open approaches according to the extent of the tumor and other patient's needs.

## CONCLUSION

Advanced endoscopic endonasal approaches provide an important addition to the surgical armamentarium for therapeutic management of the skull base malignancies. A critical review of the literature shows that it yields satisfactory outcomes and limited morbidity; however, these outcomes are highly influenced by significant selection biases regarding stage, invasiveness, and histopathology of the tumors. Therefore, proper selection of cases is critical to the achievement of excellent outcomes.
